# Climate change, environmental exposures, and breast cancer: advancing climate-resilient oncology through sustainable development goal 13

**DOI:** 10.3389/fpubh.2026.1841967

**Published:** 2026-09-11

**Authors:** Emmanuel Ifeanyi Obeagu

**Affiliations:** 1 Division of Haematology, Department of Biomedical and Laboratory Science, Africa University, Mutare, Zimbabwe; 2 The Department of Molecular Medicine and Haematology, School of Pathology, Faculty of Health Sciences, University of the Witwatersrand, Johannesburg, South Africa; 3 Department of Haematology and Immonohaematology, Bahir Dar University, Bahir Dar, Ethiopia

**Keywords:** breast cancer, cancer prevention, climate change, environmental exposures, SDG 13

## Abstract

Climate change is increasingly recognized as a major determinant of human health, yet its implications for breast cancer prevention, progression, and healthcare delivery remain insufficiently integrated into global cancer control strategies. Breast cancer, the most commonly diagnosed malignancy among women worldwide, is influenced by a complex interplay of genetic, hormonal, environmental, and socioeconomic factors, many of which are being reshaped by climate change. This narrative review examines the intersections between climate change, environmental exposures, and breast cancer within the framework of the United Nations Sustainable Development Goal 13 (Climate Action). Current evidence indicates that climate-driven environmental changes—including worsening air pollution, wildfire emissions, endocrine-disrupting chemicals, pesticide exposure, water contamination, and food system disruptions—may contribute to breast carcinogenesis through mechanisms involving oxidative stress, chronic inflammation, immune dysregulation, endocrine perturbation, and epigenetic modification. The review further highlights that susceptibility may differ across molecular subtypes, with endocrine-disrupting chemicals having greater relevance to hormone receptor-positive disease, while inflammatory and immune-mediated pathways may influence more aggressive phenotypes, although subtype-specific evidence remains limited. Beyond biological mechanisms, climate-related disasters disrupt breast cancer screening, diagnosis, treatment continuity, and survivorship care, disproportionately affecting socioeconomically disadvantaged populations through interactions with established determinants of health, including healthcare access, lifestyle factors, and structural inequities. The review critically evaluates the current alignment between SDG 13 and breast cancer control, identifying important gaps in the integration of oncology into national climate adaptation policies while highlighting opportunities for climate-resilient health systems, green oncology, exposomics, artificial intelligence, and precision environmental health.

## Introduction

Breast cancer is the most frequently diagnosed cancer and the leading cause of cancer-related mortality among women worldwide, accounting for approximately 2.3 million new cases and more than 670,000 deaths annually. Despite substantial advances in early detection, molecular characterization, targeted therapies, and multidisciplinary care, the global burden of breast cancer continues to rise, particularly in low- and middle-income countries (LMICs), where healthcare systems often face limited resources, delayed diagnosis, and unequal access to treatment. Although inherited genetic mutations such as BRCA1, BRCA2, PALB2, and TP53 contribute significantly to individual susceptibility, most breast cancers arise from complex interactions among genetic predisposition, hormonal influences, lifestyle factors, environmental exposures, and social determinants of health (1). Climate change has emerged as one of the greatest global public health challenges of the twenty-first century, extending far beyond its recognized effects on infectious diseases, food security, and environmental degradation. Increasing global temperatures, worsening air pollution, prolonged wildfire seasons, biodiversity loss, altered precipitation patterns, extreme weather events, and ecosystem disruption are reshaping patterns of environmental exposure that influence the development and progression of chronic diseases, including cancer. The Intergovernmental Panel on Climate Change (IPCC) and the World Health Organization (WHO) have emphasized that climate change represents a critical threat to human health by modifying physical, chemical, biological, and social environments. While considerable attention has focused on respiratory, cardiovascular, and infectious diseases, growing evidence indicates that climate-sensitive environmental exposures may also contribute to carcinogenesis through multiple interconnected biological pathways (2).

Environmental factors have long been recognized as important contributors to breast cancer risk. Exposure to fine particulate matter (PM₂.₅), polycyclic aromatic hydrocarbons, persistent organic pollutants, endocrine-disrupting chemicals, pesticides, heavy metals, and per- and polyfluoroalkyl substances (PFAS) has been associated with oxidative stress, chronic inflammation, endocrine disruption, DNA damage, immune dysregulation, and epigenetic modifications that may promote breast carcinogenesis. Climate change can intensify these exposures by increasing wildfire frequency, redistributing environmental contaminants through flooding, altering agricultural practices and pesticide use, accelerating plastic degradation, and worsening ambient air pollution. Rather than acting independently, climate change and environmental pollutants interact to create cumulative exposure profiles that may influence breast cancer susceptibility across the life course.

Breast cancer is a biologically heterogeneous disease comprising distinct molecular subtypes with different etiologies, prognoses, and therapeutic responses. Hormone receptor-positive tumors, HER2-positive cancers, and triple-negative breast cancers exhibit unique molecular characteristics that may influence their susceptibility to environmental exposures. For example, endocrine-disrupting chemicals may preferentially affect estrogen receptor-positive disease through hormonal signaling pathways, whereas oxidative stress, inflammatory signaling, and immune dysfunction may contribute to more aggressive phenotypes such as triple-negative breast cancer. Although evidence supporting subtype-specific environmental effects continues to evolve, recognizing this heterogeneity is essential for developing precision prevention strategies and identifying vulnerable populations (3). Beyond its biological effects, climate change increasingly threatens the delivery of equitable cancer care. Heatwaves, floods, hurricanes, droughts, and wildfires can disrupt cancer screening programs, damage healthcare infrastructure, interrupt pathology and imaging services, delay surgery and systemic therapies, compromise pharmaceutical supply chains, and reduce access to follow-up care. These disruptions disproportionately affect populations already experiencing social and economic disadvantage, including rural communities, indigenous populations, racial and ethnic minorities, and residents of LMICs. However, disparities in breast cancer outcomes arise from multiple interacting determinants, including obesity, tobacco use, alcohol consumption, reproductive history, healthcare accessibility, screening participation, socioeconomic status, and structural inequities within healthcare systems. Climate-related environmental exposures should therefore be viewed as one component of a broader network of determinants that collectively shape breast cancer risk and outcomes (4).

The United Nations Sustainable Development Goal 13 (SDG 13) calls for urgent action to combat climate change and strengthen adaptation and resilience across all sectors. Although SDG 13 has primarily focused on reducing greenhouse gas emissions, disaster preparedness, and environmental sustainability, its implementation has important implications for non-communicable diseases, including cancer. Climate-resilient health systems, sustainable healthcare infrastructure, environmental pollution control, disaster preparedness, and intersectoral climate governance can indirectly support breast cancer prevention, improve continuity of oncology services during climate-related emergencies, and reduce environmentally mediated health risks. Nevertheless, breast cancer and other non-communicable diseases remain underrepresented in many national climate adaptation strategies and Nationally Determined Contributions (NDCs), highlighting an important policy gap that warrants greater attention (5). Recent advances in exposomics, geospatial epidemiology, artificial intelligence, remote sensing, environmental monitoring, and multi-omics technologies have created new opportunities to better characterize the complex relationships between climate change, environmental exposures, and breast cancer. Integrating these emerging approaches with precision oncology may facilitate identification of high-risk populations, improve environmental risk prediction, strengthen surveillance systems, and inform climate-responsive cancer prevention strategies (6). This narrative review critically examines current evidence on the intersections between climate change, environmental exposures, and breast cancer within the framework of SDG 13. Rather than simply summarizing individual environmental risk factors, the review synthesizes how climate change modifies environmental exposures, biological mechanisms, healthcare delivery, and health equity to influence breast cancer across the continuum of prevention, diagnosis, treatment, and survivorship. Furthermore, it identifies knowledge gaps, evaluates the extent to which climate policies currently address breast cancer and other non-communicable diseases, and proposes future research and policy priorities for developing climate-resilient, equitable, and sustainable oncology systems in an era of accelerating environmental change.

## Aim

The aim of this narrative review is to critically examine the complex interactions between climate change, environmental exposures, and breast cancer within the framework of the United Nations Sustainable Development Goal 13 (Climate Action). Specifically, the review synthesizes current evidence on the biological, environmental, and health system pathways through which climate change may influence breast cancer risk, molecular heterogeneity, disease progression, healthcare delivery, and survivorship. It further evaluates the extent to which SDG 13 and existing climate adaptation policies address breast cancer and other non-communicable diseases, identifies current knowledge gaps, and highlights opportunities for climate-resilient oncology, precision environmental health, and sustainable policy interventions to advance equitable breast cancer prevention and care in a changing global landscape.

## Methods

### Literature search and narrative review design

This article was developed as a structured narrative review examining the relationships among climate change, environmental exposures, breast cancer risk and biology, clinical outcomes, and the development of climate-resilient oncology within the framework of Sustainable Development Goal 13 (SDG 13: Climate Action). The review was designed to provide an integrated synthesis of evidence from environmental health, cancer epidemiology, molecular oncology, climate science, public health, and health-system research rather than to constitute a formally registered systematic review or meta-analysis.

### Literature identification

A structured literature search was conducted using major biomedical and multidisciplinary sources, including PubMed/MEDLINE, Scopus, Web of Science, Google Scholar, and relevant reports from international organizations. Search concepts were developed around four principal domains: (i) climate change and climate-related hazards; (ii) environmental exposures; (iii) breast cancer; and (iv) climate-resilient cancer prevention and healthcare. Search terms and their combinations included, but were not limited to, *“climate change,” “global warming,” “environmental exposure,” “environmental pollutants,” “air pollution,” “PM2.5,” “wildfire smoke,” “polycyclic aromatic hydrocarbons,” “endocrine-disrupting chemicals,” “persistent organic pollutants,” “pesticides,” “heavy metals,” “heat exposure,” “flooding,” “breast cancer,” “breast carcinogenesis,” “breast cancer progression,” “breast cancer outcomes,” “cancer prevention,” “health-system resilience,”* and *“climate-resilient oncology.”* Boolean operators were used to combine related concepts where appropriate.

### Eligibility and selection of evidence

Literature was considered eligible when it provided substantive information relevant to one or more of the following areas: environmental determinants of breast cancer; climate-sensitive environmental exposures; biological mechanisms potentially linking environmental exposures with breast carcinogenesis; climate-related effects on breast cancer diagnosis, treatment, or outcomes; environmental and social inequities; or strategies for developing climate-resilient and environmentally sustainable oncology services. Peer-reviewed original research, epidemiological studies, mechanistic investigations, systematic and narrative reviews, authoritative scientific assessments, and relevant reports from recognized international organizations were considered. Publications were excluded when they lacked substantive relevance to the review objectives, duplicated evidence without providing additional interpretive value, or did not contain sufficient information to support meaningful synthesis. Particular attention was given to evidence addressing air pollution, particulate matter, polycyclic aromatic hydrocarbons, endocrine-disrupting chemicals, persistent organic pollutants, pesticides, heavy metals, wildfire exposure, extreme heat, food-system disruption, and climate-related healthcare interruptions.

### Screening and evidence synthesis

Retrieved literature was assessed for relevance based on title and abstract information, followed by full-text consideration where appropriate. The selection process was iterative and thematic, consistent with the objectives of a narrative review. Articles providing complementary epidemiological, mechanistic, clinical, environmental, or policy perspectives were retained to facilitate integration across disciplines. The final documented evidence base comprised 65 articles. Because the review was not prospectively registered as a systematic review and contemporaneous records were not available to independently verify all historical intermediate screening counts, specific numerical counts for database retrievals, duplicates, records screened, and full-text exclusions are not reported. This approach was adopted deliberately to avoid presenting retrospectively reconstructed values as prospectively documented screening data.

### Assessment and interpretation of evidence

The evidence was critically interpreted according to the strength and nature of the available evidence. Particular care was taken to distinguish established associations, biologically plausible mechanisms, emerging evidence, and hypotheses requiring further investigation. The review does not assume that climate change is itself a direct breast carcinogen. Instead, climate change is conceptualized as a risk modifier, exposure amplifier, and health-system stressor capable of altering the distribution and intensity of environmental hazards and disrupting cancer prevention and treatment. Where evidence was observational or mechanistic, causal conclusions were avoided unless adequately supported. The review also recognized potential confounding, exposure misclassification, differences in exposure assessment, geographical heterogeneity, and the long latency between environmental exposure and breast cancer development.

### Conceptual integration

The review used an integrated Climate–Exposome–Breast Cancer–Resilience framework to connect environmental and climatic processes with biological susceptibility and healthcare outcomes. This framework considers climate change as an upstream determinant that can modify environmental exposures, including air pollutants, persistent chemicals, pesticides, heavy metals, heat, and wildfire-related contaminants. These exposures may converge through oxidative stress, chronic inflammation, endocrine disruption, epigenetic alterations, genomic instability, immune dysregulation, and metabolic dysfunction. The framework further recognizes that climate change can affect breast cancer outcomes independently of tumor initiation by disrupting screening, diagnostic pathways, treatment continuity, pharmaceutical supply chains, energy and water availability, transportation, and survivorship services.

### Methodological scope and limitations

This review provides a structured narrative synthesis rather than a quantitative systematic review. Accordingly, no meta-analysis, pooled effect estimates, formal risk-of-bias scoring, or statistical assessment of publication bias was undertaken. The heterogeneous nature of the evidence—including differences in exposure definitions, study populations, geographical settings, outcome measures, and biological endpoints—also limits direct quantitative comparison across studies. The review therefore emphasizes convergence of evidence across epidemiological, mechanistic, environmental, and health-system domains rather than assigning numerical certainty to every proposed climate–breast cancer relationship. The final evidence base of 65 articles represents the documented literature selected as most relevant to the scope and objectives of the review.

### Ethical considerations

As this review involved analysis of previously published literature and publicly available reports and did not involve direct recruitment of human participants or access to identifiable patient-level data, ethical approval was not required.

## Climate change, environmental stressors, and breast cancer risk

Climate change is increasingly recognized as an upstream determinant of environmental health that modifies the distribution, intensity, and persistence of multiple breast cancer risk factors. Rather than acting as a direct carcinogen, climate change alters physical, chemical, and biological environments in ways that influence human exposure to established and emerging carcinogenic agents. Rising global temperatures, extreme weather events, prolonged droughts, flooding, wildfires, ecosystem degradation, and changing agricultural practices collectively reshape exposure profiles throughout the life course, potentially contributing to breast carcinogenesis through complex and interrelated mechanisms (7, 8). One of the most extensively studied climate-sensitive environmental stressors is ambient air pollution. Increasing temperatures accelerate ground-level ozone formation, while wildfires and prolonged droughts elevate concentrations of fine particulate matter (PM₂.₅), polycyclic aromatic hydrocarbons (PAHs), volatile organic compounds, and heavy metals. Long-term exposure to these pollutants has been associated with increased breast cancer incidence in several epidemiological studies, particularly among postmenopausal women. Mechanistically, inhaled pollutants induce oxidative stress, DNA damage, chronic systemic inflammation, mitochondrial dysfunction, and epigenetic alterations that may promote malignant transformation of mammary epithelial cells. Although associations vary across studies, meta-analyses generally support a modest increase in breast cancer risk associated with prolonged exposure to PM₂.₅ and nitrogen dioxide (NO₂), while emphasizing the need for improved exposure assessment and longitudinal investigation (9, 10).

Climate change also influences exposure to endocrine-disrupting chemicals (EDCs), including bisphenol A (BPA), phthalates, organochlorine pesticides, dioxins, polychlorinated biphenyls (PCBs), and per- and polyfluoroalkyl substances (PFAS). Environmental degradation, flooding, soil erosion, increased plastic waste, and altered industrial emissions may redistribute these persistent contaminants within ecosystems and drinking water supplies. Many EDCs exhibit estrogenic or anti-estrogenic properties, disrupt endocrine homeostasis, and interfere with mammary gland development, making them biologically plausible contributors to hormone-dependent breast carcinogenesis. Experimental studies demonstrate that these compounds can activate estrogen receptor signaling, alter growth factor pathways, induce epigenetic modifications, and promote cellular proliferation. However, epidemiological evidence remains heterogeneous owing to differences in exposure timing, dose, and study design (11, 12). Climate-related alterations in food systems may further influence breast cancer risk. Rising temperatures, reduced agricultural productivity, and increased dependence on pesticide-intensive farming may elevate dietary exposure to agricultural chemicals while simultaneously affecting nutritional quality and food security. Climate change also contributes indirectly to obesity through changes in food availability, dietary patterns, and reduced opportunities for physical activity during extreme heat events. Since obesity is an established risk factor for postmenopausal breast cancer through increased aromatase activity, chronic inflammation, insulin resistance, and elevated circulating estrogen concentrations, climate-driven changes in food environments may indirectly contribute to breast cancer incidence (13).

Wildfires have become increasingly frequent and severe in many regions due to climate change, generating complex mixtures of carcinogenic compounds including benzene, formaldehyde, PAHs, ultrafine particulate matter, and heavy metals. Recurrent wildfire smoke exposure has been associated with increased systemic inflammation and oxidative stress, although direct epidemiological evidence linking wildfire exposure to breast cancer remains limited. Current findings should therefore be interpreted cautiously, and additional prospective studies are needed to determine long-term cancer risks associated with repeated wildfire events (14). Climate-induced flooding and water contamination represent additional pathways through which environmental exposures may be altered. Floodwaters can mobilize industrial pollutants, pesticides, pharmaceutical residues, heavy metals, and PFAS into surface and groundwater sources, increasing opportunities for chronic human exposure. Although direct causal evidence linking contaminated water resulting from climate events to breast cancer is limited, several contaminants identified in affected water systems have demonstrated endocrine-disrupting or carcinogenic properties in experimental models (15). Occupational environmental exposures may also change under a warming climate. Agricultural workers, firefighters, industrial employees, and waste management personnel may experience increased exposure to pesticides, combustion products, diesel exhaust, solvents, and heat stress. These occupational hazards may interact with environmental pollution to produce cumulative lifetime exposures that influence cancer susceptibility. Women employed in these occupations may represent understudied populations requiring greater attention in environmental epidemiology (16). Breast cancer is a biologically heterogeneous disease, and environmental stressors may not affect all molecular subtypes equally. Current evidence suggests that endocrine-disrupting chemicals may exert greater influence on estrogen receptor-positive (ER-positive) tumors through hormone receptor-mediated signaling pathways. In contrast, oxidative stress, inflammatory mediators, immune dysregulation, and environmental toxicants that induce genomic instability may contribute to the development or progression of more aggressive subtypes, including triple-negative breast cancer (TNBC), although definitive subtype-specific associations remain incompletely established. Similarly, susceptibility may vary according to menopausal status, genetic predisposition, reproductive history, and cumulative lifetime exposure, emphasizing the importance of precision environmental health approaches in future research (6, 17).

### Climate-driven modifiers of breast cancer progression and outcomes

Climate change may influence breast cancer outcomes through a combination of environmental, biological, behavioral, and health-system pathways. However, the available evidence does not establish climate change itself as a direct cause of breast cancer progression or metastasis. Rather, climate-related conditions may modify exposures and physiological or social environments that are relevant to tumor biology and clinical outcomes. Increasing ambient temperatures and recurrent heatwaves may impose physiological stress that affects oxidative balance, inflammation, metabolism, immune function, and endocrine regulation. These pathways are biologically relevant to breast cancer because chronic inflammation, oxidative stress, metabolic alterations, and immune dysregulation can participate in tumor development and progression (16). Experimental and mechanistic evidence provides a rationale for investigating whether sustained heat exposure can influence the tumor microenvironment. Heat-related physiological stress may potentially affect inflammatory signaling, cellular stress responses, angiogenic pathways, and immune-cell activity. Nevertheless, direct evidence demonstrating that environmental heat exposure promotes breast cancer growth or metastasis in humans remains limited. Therefore, these mechanisms should currently be regarded as plausible pathways requiring prospective epidemiological and translational investigation rather than established causal relationships (17).

Climate change can alter the concentration, composition, and geographic distribution of air pollutants through increased wildfire activity, higher temperatures, altered atmospheric circulation, and changes in energy use. Exposure to particulate matter, nitrogen dioxide, ozone, PAHs, and other combustion-related pollutants may contribute to oxidative stress, systemic inflammation, DNA damage, and endocrine or immune perturbation (6). These mechanisms may affect biological pathways relevant to tumor progression and treatment response. However, the strength of evidence differs among pollutants, and associations should not automatically be interpreted as evidence that climate change directly drives breast cancer progression. Further longitudinal studies incorporating individual exposure measurements and tumor molecular characteristics are needed (18). Wildfire smoke contains fine particulate matter and multiple potentially toxic compounds, including PAHs, volatile organic compounds, and other combustion products. As climate change contributes to conditions conducive to more frequent or intense wildfires in some regions, populations may experience greater cumulative exposure (19). Wildfire-related pollutants may contribute to oxidative and inflammatory processes that are relevant to cancer biology. However, evidence directly linking repeated wildfire-smoke exposure with breast cancer progression or metastasis remains insufficient. Future studies should investigate cumulative exposure, molecular tumor characteristics, treatment response, and long-term outcomes (20).

Climate-related environmental changes may modify the distribution, persistence, transport, and human exposure to endocrine-disrupting chemicals (EDCs), including certain pesticides, PFAS, bisphenols, phthalates, and persistent organic pollutants. Because breast tissue is highly responsive to hormonal signaling, disruption of estrogenic and other endocrine pathways may potentially affect cellular proliferation and other processes relevant to carcinogenesis (21). For patients with hormone-receptor-positive breast cancer, environmental endocrine disruption represents an important research question. Nevertheless, evidence remains heterogeneous, and the clinical significance of environmental EDC exposure during established breast cancer requires further investigation (22). Several climate-related exposures converge on common biological pathways, particularly oxidative stress and chronic inflammation. Air pollution, wildfire smoke, chemical exposures, extreme heat, and psychosocial stress may contribute to inflammatory or oxidative processes. These processes may influence the tumor microenvironment by affecting immune-cell recruitment, cytokine signaling, angiogenesis, extracellular-matrix remodeling, and cellular metabolism. Such effects could theoretically modify tumor behavior or treatment response. However, the extent to which climate-related exposures produce clinically meaningful changes in the breast cancer microenvironment remains uncertain (23).

The strongest and most immediate evidence for climate-related effects on breast cancer outcomes may arise through health-system disruption rather than direct tumor biology. Floods, heatwaves, wildfires, storms, droughts, and other extreme events can interrupt screening, diagnostic imaging, pathology, surgery, radiotherapy, chemotherapy, medication supply, transportation, and follow-up care. Treatment delays or interruptions may subsequently contribute to disease progression through established clinical pathways. Climate change may therefore indirectly affect breast cancer outcomes by reducing the timeliness, continuity, and quality of cancer care (24). Climate-related disasters may increase psychological stress, displacement, financial insecurity, food insecurity, and barriers to healthcare access. These factors can affect treatment adherence, nutritional status, physical activity, mental health, and continuity of follow-up. The consequences may be particularly pronounced among populations already experiencing socioeconomic disadvantage. Climate change may therefore amplify existing breast cancer inequalities rather than acting as an isolated biological determinant (Table 1) (25, 26).

**Table 1:** Climate-driven modifiers of breast cancer progression and outcomes.

Climate-related modifier	Exposure or pathway	Potential relevance to breast cancer progression/outcomes	Key biological/clinical mechanism	Populations/settings of concern	Evidence type and strength	Key limitations
Ambient air pollution	Climate-sensitive changes in PM₂.₅, PM₁₀, NO₂, ozone and combustion pollutants	May be associated with breast cancer incidence and potentially poorer outcomes; evidence for progression is less established	Oxidative stress, systemic inflammation, DNA damage, epigenetic alterations and immune dysregulation	Urban populations, traffic-exposed communities, populations near industrial sources	Moderate epidemiological evidence for incidence; limited/emerging evidence for progression and survival	Residual confounding, exposure misclassification and difficulty separating climate effects from baseline pollution
Wildfire smoke	PM₂.₅, PAHs, benzene and other combustion-derived pollutants	Repeated exposure may affect pathways relevant to carcinogenesis and potentially treatment outcomes, but direct breast cancer evidence remains limited	Oxidative stress, inflammatory signaling, DNA damage and altered immune responses	Wildfire-prone regions, firefighters and communities with recurrent smoke exposure	Strong mechanistic plausibility; limited direct epidemiological evidence	Few breast cancer-specific longitudinal studies; complex smoke composition and variable exposure duration
Extreme heat	Prolonged high temperatures and heatwaves	May indirectly worsen outcomes by disrupting treatment, increasing physiological stress and reducing healthcare accessibility	Dehydration, cardiovascular stress, treatment intolerance, infrastructure failure and healthcare disruption	Older patients, individuals with comorbidities, outdoor workers and poorly resourced settings	Moderate evidence for health-service disruption; insufficient evidence for direct tumor progression	Direct effects on human breast tumor biology remain poorly established
Flooding and extreme precipitation	Mobilization of industrial chemicals, pesticides, sewage and persistent contaminants	May increase exposure to hazardous chemicals and interrupt cancer diagnosis and treatment	Contaminant redistribution, displacement, infrastructure damage, treatment interruption and psychological stress	Flood-prone communities and settings with fragile water and health systems	Moderate evidence for healthcare disruption; limited evidence linking flood-related exposures directly to breast cancer	Long cancer latency and difficulties reconstructing historical exposure
Climate-modified endocrine-disrupting chemical exposure	Redistribution or altered exposure to BPA, phthalates, PCBs, PFAS and other EDCs	May be particularly relevant to hormone-sensitive breast carcinogenesis; effects on progression require further study	Estrogen receptor signaling, altered hormone metabolism, epigenetic regulation and proliferative signaling	Highly exposed occupational or environmentally contaminated populations	Moderate mechanistic evidence; heterogeneous epidemiological evidence	EDCs are not created by climate change; climate change may modify their distribution, persistence and exposure patterns
Climate-sensitive pesticide exposure	Changes in pest distribution and agricultural pesticide application	Certain compounds may be associated with breast cancer risk, particularly following prolonged or critical-window exposure	Endocrine disruption, oxidative stress, genotoxicity and epigenetic modification	Agricultural workers and rural communities	Moderate mechanistic but mixed epidemiological evidence	Heterogeneity between pesticide classes, co-exposures and limited subtype-specific evidence
Water contamination	Climate-related mobilization of PFAS, metals, pesticides and industrial pollutants	May modify cumulative exposure to compounds with endocrine-disrupting or carcinogenic potential	Endocrine perturbation, oxidative stress, genotoxicity and epigenetic changes	Flood-prone areas and communities dependent on vulnerable water supplies	Emerging/limited breast cancer-specific evidence	Attribution to climate change is difficult; exposure often precedes climate-related redistribution
Food-system disruption	Drought, crop failure, food insecurity and altered food environments	May indirectly influence breast cancer risk and survivorship through metabolic and nutritional pathways	Obesity, insulin resistance, inflammation and altered estrogen metabolism	Food-insecure populations and climate-vulnerable LMICs	Strong evidence for obesity as a breast cancer determinant; indirect evidence for climate-mediated pathway	Multiple socioeconomic and behavioral confounders
Occupational climate exposure	Heat, wildfire smoke, pesticides, diesel emissions and industrial chemicals	May increase cumulative environmental exposure among selected occupational groups	Oxidative stress, endocrine disruption, inflammation and genotoxicity	Agricultural workers, firefighters, industrial workers and outdoor workers	Variable; exposure-specific epidemiological and mechanistic evidence	Healthy-worker effects, mixed exposures and inadequate sex-specific longitudinal data
Extreme-weather healthcare disruption	Damage to hospitals, transport systems, laboratories, energy supplies and pharmaceutical supply chains	Can delay screening, diagnosis, surgery, systemic therapy and radiotherapy, potentially worsening outcomes	Treatment interruption, delayed diagnosis, medication shortages and reduced follow-up	Patients in disaster-prone regions, rural communities and resource-constrained health systems	Moderate-to-strong evidence for service disruption; outcome evidence varies	Difficult to separate disaster effects from baseline healthcare inequalities
Climate-related displacement	Migration, evacuation and loss of continuity of care	May contribute to delayed diagnosis, treatment interruption and loss to follow-up	Healthcare fragmentation, financial hardship, psychosocial stress and disrupted medical records	Displaced populations, refugees and socioeconomically vulnerable patients	Moderate evidence for healthcare access disruption; limited breast cancer-specific longitudinal evidence	Substantial socioeconomic and healthcare-system confounding
Energy and infrastructure instability	Power outages and damage to health facilities during extreme events	May compromise imaging, pathology, drug storage, surgery and radiotherapy	Diagnostic delays, interruption of temperature-sensitive medicines and reduced treatment capacity	Low-resource and disaster-exposed oncology centers	Moderate health-system evidence	Few studies directly quantify effects on breast cancer-specific survival
Cumulative climate–social vulnerability	Interaction of environmental hazards with poverty, housing, comorbidities and limited healthcare access	May amplify disparities in stage at diagnosis, treatment continuity and survival	Combined environmental, behavioral, socioeconomic and structural pathways	Marginalized and underserved populations	Strong evidence for social determinants; emerging evidence for climate interaction	High potential for confounding; climate exposure should not be treated as the sole cause of disparities

BPA, bisphenol A; EDCs, endocrine-disrupting chemicals; LMICs, low- and middle-income countries; NO₂, nitrogen dioxide; PAHs, polycyclic aromatic hydrocarbons; PCBs, polychlorinated biphenyls; PFAS, per- and polyfluoroalkyl substances; PM, particulate matter.

### Environmental inequities and climate-driven disparities in breast cancer

Environmental inequities are a long-standing driver of disparities in breast cancer incidence and outcomes, and climate change is intensifying these vulnerabilities across regions and populations. As environmental conditions become more volatile and pollutant burdens increase, women who already face socioeconomic disadvantage, limited healthcare access, and structural marginalization bear a disproportionate share of risk. The intersection of climate change with pre-existing environmental injustices creates a compounded effect: higher exposure to carcinogenic stressors, greater barriers to early detection and treatment, and poorer survivorship outcomes (27, 28). In many urban and industrial regions, marginalized communities are more likely to live near pollution sources such as refineries, manufacturing zones, waste-processing facilities, and congested traffic corridors. These environments often have elevated levels of particulate matter, polycyclic aromatic hydrocarbons, heavy metals, and endocrine-disrupting chemicals, all of which are associated with increased breast cancer risk. Climate change exacerbates these exposures through mechanisms such as heat-driven volatilization of chemicals, worsening air stagnation, and more frequent wildfire smoke events. As a result, communities already facing environmental burdens experience intensified carcinogenic exposure profiles, reinforcing geographic and social gradients in breast cancer susceptibility (29, 30).

Rural and agricultural regions face a different but equally concerning set of inequities. Women in these settings often experience chronic exposure to agricultural pesticides, fertilizers, and other endocrine-disrupting agents whose distribution is influenced by climate patterns. Rising temperatures and shifting pest populations can increase pesticide use, while droughts and floods alter chemical mobility in soil and water systems. Limited regulatory oversight and insufficient environmental monitoring in many low- and middle-income settings further heighten risk. The combined effect is a disproportionate toxic burden that may elevate long-term breast cancer risk among women with occupational or residential exposure (31, 32). Healthcare access—already unequally distributed across socioeconomic strata—becomes even more fragile under climate stress. Communities with limited healthcare infrastructure are often the first to experience service disruptions during extreme weather events. Floods, storms, and heatwaves can restrict transportation, damage diagnostic facilities, and interrupt screening programs. These disruptions disproportionately affect women who rely on public services, live far from tertiary care centers, or lack the financial means to seek private care. Consequently, delayed diagnosis, later-stage presentation, and inconsistent treatment adherence become more prevalent in climate-vulnerable populations, driving poorer survival outcomes (33).

Infrastructure disparities also extend to the availability of climate-resilient health systems. High-income countries are better positioned to invest in backup power systems, resilient supply chains, and redundant care pathways, whereas low-resource regions struggle to maintain continuity of care during climate emergencies. This imbalance magnifies global inequities in breast cancer outcomes, as delays in chemotherapy, radiotherapy, or surgical management can substantially reduce prognosis. Furthermore, disruptions in cold-chain systems required for storing targeted therapies, hormonal agents, and diagnostic reagents pose significant risks in settings with unstable electricity grids (34, 35). Social determinants of health—income, education, housing stability, and occupational conditions—intersect closely with climate-driven disparities. Women in informal settlements or substandard housing often experience higher exposure to extreme heat, poor indoor air quality, and flood-related contamination. These environmental stressors compound psychosocial burdens such as financial insecurity and displacement, which may reduce treatment adherence and affect physiological stress responses relevant to cancer progression. Climate-related displacement—from both sudden disasters and slow-onset environmental degradation—further limits continuity of care for breast cancer survivors and exacerbates long-term disparities (36, 37).

In addition, global climate change reinforces inequities across demographic lines, including racial and ethnic minorities who disproportionately reside in environmentally hazardous areas due to historical segregation, discriminatory urban planning, and unequal economic opportunity. These communities face compounded vulnerability: higher exposure to environmental carcinogens, climate-sensitive infrastructure failures, and reduced access to high-quality oncology services. As climate change accelerates, these structural inequities widen, producing a disproportionate burden of advanced disease and poorer outcomes in marginalized populations (Table 2) (38, 39).

**Table 2:** Environmental inequities and climate-driven disparities in breast cancer.

Population or setting	Climate-related environmental challenge	Interaction with established determinants of breast cancer	Potential impact on breast cancer outcomes	Evidence type and strength	Priority SDG 13 actions
Low-income communities	Higher exposure to air pollution, industrial emissions, extreme heat, and flooding	Poverty, obesity, limited education, unhealthy diets, reduced healthcare access	Delayed diagnosis, advanced-stage disease, poorer survival, treatment interruption	Moderate epidemiological evidence; strong evidence for socioeconomic disparities	Strengthen climate-resilient primary healthcare, pollution reduction policies, and equitable adaptation financing
Rural populations	Wildfires, droughts, water scarcity, transportation disruption	Limited screening services, workforce shortages, geographic isolation	Reduced mammography uptake, delayed diagnosis, interrupted treatment	Moderate evidence from health services research	Expand decentralized oncology, telemedicine, resilient transport systems, and mobile screening programs
Women in low- and middle-income countries (LMICs)	Climate-sensitive food insecurity, flooding, heatwaves, healthcare infrastructure vulnerability	Late presentation, limited diagnostic capacity, financial barriers, inadequate oncology services	Higher mortality, delayed treatment initiation, reduced treatment completion	Strong evidence for healthcare inequities; emerging evidence for climate interactions	Integrate cancer services into national climate adaptation plans and strengthen resilient health systems
Racial and ethnic minority populations	Disproportionate residence near polluted environments and climate-vulnerable areas	Structural racism, lower screening uptake, insurance disparities, healthcare bias	Increased stage at diagnosis, treatment disparities, poorer survival	Strong epidemiological evidence for health disparities; emerging evidence for climate amplification	Promote environmental justice, equitable healthcare access, and targeted adaptation policies
Indigenous populations	Ecosystem degradation, biodiversity loss, water insecurity, environmental contamination	Geographic isolation, cultural barriers, limited specialist services	Delayed diagnosis, lower treatment access, poorer continuity of care	Moderate evidence	Community-led climate adaptation, culturally appropriate cancer prevention, and improved service accessibility
Older women	Heatwaves, climate-related disasters, disrupted health services	Comorbidities, reduced mobility, frailty, polypharmacy	Increased treatment delays, reduced treatment tolerance, interrupted follow-up	Moderate clinical evidence	Climate-resilient geriatric oncology services and emergency preparedness
Occupationally exposed women (agriculture, firefighting, industry)	Heat stress, pesticides, combustion products, diesel exhaust, wildfire smoke	Long-term occupational exposure, variable use of protective equipment	Increased cumulative environmental exposure; possible elevated breast cancer risk in selected occupations	Mixed epidemiological and mechanistic evidence	Strengthen occupational safety regulations, exposure monitoring, and climate-adaptive workplace policies
Displaced populations and climate migrants	Forced migration following floods, droughts, storms, or conflict	Loss of medical records, interrupted care, financial hardship, unstable housing	Delayed diagnosis, discontinuity of treatment, reduced survivorship care	Emerging evidence	Develop portable health records, continuity-of-care systems, and inclusive disaster-response planning
Urban populations in highly polluted cities	Persistent air pollution exacerbated by heat and climate-driven atmospheric changes	Sedentary lifestyles, obesity, socioeconomic inequality	Increased cumulative exposure to environmental pollutants; potential adverse effects on breast cancer risk	Moderate epidemiological evidence	Improve urban air quality, reduce emissions, expand green infrastructure, and promote sustainable transport
Communities affected by recurrent flooding	Water contamination with pesticides, heavy metals, PFAS, and industrial pollutants	Limited clean water, poor sanitation, constrained healthcare access	Potential long-term environmental exposure and disruption of cancer services	Limited direct breast cancer evidence; moderate environmental evidence	Strengthen water safety, environmental monitoring, and resilient healthcare infrastructure
Women experiencing food insecurity	Climate-driven crop failures, rising food costs, reduced dietary quality	Obesity, malnutrition, metabolic disorders, reduced preventive healthcare utilization	Indirect influence on breast cancer risk through metabolic and inflammatory pathways	Strong evidence for obesity–breast cancer relationship; indirect evidence for climate pathway	Promote climate-smart agriculture, nutrition programs, and food security initiatives
Healthcare systems in climate-vulnerable regions	Damage to hospitals, power outages, disrupted pharmaceutical supply chains	Limited oncology workforce, resource constraints, inadequate emergency preparedness	Delayed surgery, interrupted chemotherapy and radiotherapy, compromised diagnostic capacity	Strong health systems evidence	Invest in climate-resilient oncology infrastructure, renewable energy, digital health, and disaster preparedness

LMICs, low- and middle-income countries; PFAS, per- and polyfluoroalkyl substances; SDG 13, Sustainable Development Goal 13.

### Policy, prevention, and the SDG 13 framework

The growing recognition of climate change as a determinant of non-communicable diseases has created an opportunity to integrate environmental sustainability into comprehensive breast cancer prevention and control. The United Nations Sustainable Development Goal 13 (SDG 13) calls for urgent action to combat climate change and its impacts through mitigation, adaptation, resilience building, climate governance, and international cooperation. Although SDG 13 was not developed specifically to address cancer, its objectives provide an important policy framework for reducing environmental risk factors, strengthening climate-resilient health systems, and protecting vulnerable populations from the health consequences of climate change. Integrating breast cancer prevention into climate action aligns with the broader One Health and Planetary Health approaches, which recognize the interconnectedness of environmental integrity, ecosystem health, and human well-being (40, 41). Current evidence suggests that many climate-sensitive environmental exposures associated with breast carcinogenesis—including ambient air pollution, endocrine-disrupting chemicals, persistent organic pollutants, wildfire emissions, contaminated water, and food system disruptions—are potentially modifiable through environmental regulation and climate adaptation strategies. Policies aimed at reducing greenhouse gas emissions, transitioning to clean energy, improving urban air quality, strengthening industrial emission standards, reducing plastic pollution, promoting sustainable agriculture, and protecting water resources may simultaneously decrease exposure to environmental carcinogens while delivering broader public health benefits. These interventions illustrate how climate mitigation can generate important co-benefits for cancer prevention beyond reductions in climate-related morbidity and mortality (42).

Climate adaptation policies are equally important for ensuring continuity of breast cancer care. Increasing frequency and intensity of floods, hurricanes, droughts, heatwaves, and wildfires threaten healthcare infrastructure, interrupt pharmaceutical supply chains, delay diagnostic services, and reduce access to surgery, radiotherapy, systemic treatment, and survivorship care. Strengthening climate-resilient health systems therefore requires investment in disaster preparedness, resilient healthcare infrastructure, renewable energy systems for hospitals, digital health technologies, teleoncology, decentralized cancer services, electronic health records, and emergency supply chain management. Such measures can reduce treatment interruptions and improve continuity of care during climate-related emergencies (43). Despite these opportunities, breast cancer and other non-communicable diseases remain inadequately represented within global climate policy. Analysis of Nationally Determined Contributions (NDCs) submitted under the Paris Agreement demonstrates that although many countries recognize the health impacts of climate change, explicit references to cancer prevention, oncology services, or environmentally related carcinogenesis remain uncommon. Similarly, national climate adaptation plans frequently prioritize infectious diseases, nutrition, water security, and disaster response while giving comparatively limited attention to chronic diseases, including breast cancer. This policy gap highlights the need for greater integration of oncology into national and international climate adaptation strategies (44).

The World Health Organization (WHO) has emphasized the importance of climate-resilient and environmentally sustainable health systems capable of maintaining essential health services during environmental emergencies. Applying these principles to breast cancer control includes protecting screening programs, maintaining pathology and diagnostic imaging services, ensuring uninterrupted access to anticancer medicines, safeguarding radiotherapy facilities, strengthening workforce preparedness, and developing contingency plans for climate-related disasters. Incorporating oncology into climate resilience planning can improve health system preparedness while reducing disparities in cancer outcomes during environmental crises (39). Prevention strategies should also adopt a broader environmental health perspective. Primary prevention should extend beyond individual behavioral modification to include reduction of environmental carcinogen exposure through strengthened regulation of industrial emissions, pesticide use, plastic waste, hazardous chemicals, and occupational hazards. Public health campaigns promoting healthy diets, physical activity, tobacco cessation, reduced alcohol consumption, and obesity prevention remain essential because these established risk factors interact with environmental and climate-related determinants rather than acting independently. Consequently, effective breast cancer prevention requires integrated interventions addressing environmental, behavioral, biological, and social determinants simultaneously (45).

Environmental justice represents another critical component of climate-responsive breast cancer policy. Climate change disproportionately affects populations with limited adaptive capacity, including low-income communities, rural populations, Indigenous peoples, racial and ethnic minorities, and residents of low- and middle-income countries. These populations often experience greater exposure to environmental pollutants while simultaneously facing barriers to screening, diagnosis, treatment, and survivorship care. However, disparities in breast cancer outcomes arise from multiple interacting factors, including socioeconomic deprivation, reproductive characteristics, obesity, healthcare accessibility, structural discrimination, and differences in screening participation. Climate-related environmental exposures should therefore be understood as amplifying existing inequities rather than serving as their sole cause. Policies that promote equitable access to preventive services, universal health coverage, environmental protection, and climate adaptation are likely to produce substantial benefits for breast cancer control (46). Emerging technologies provide additional opportunities to strengthen policy implementation. Artificial intelligence (AI), geographic information systems (GIS), satellite-based environmental monitoring, remote sensing, and exposomics can identify geographic hotspots of environmental risk, support climate-informed cancer surveillance, improve exposure assessment, and guide resource allocation. Integrating these technologies with population-based cancer registries and environmental monitoring systems may facilitate precision public health approaches capable of identifying high-risk populations and informing targeted interventions. Nevertheless, ethical considerations regarding data privacy, equitable access, algorithmic bias, and infrastructure disparities must be addressed before widespread implementation (47).

**Figure 1:**
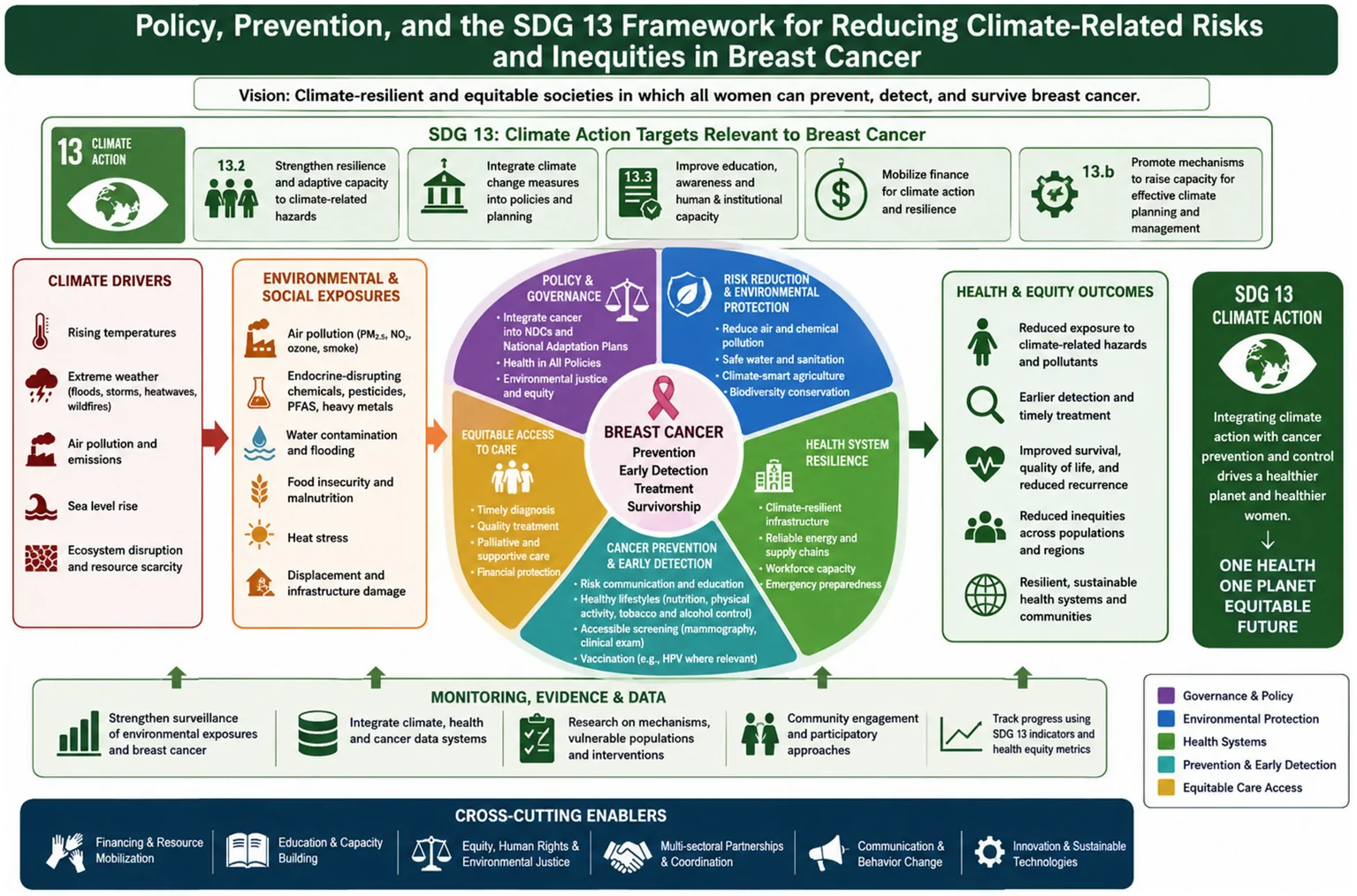
Policy, prevention, and the SDG 13 framework.

Sustainable oncology, often referred to as green oncology, has become an increasingly important component of climate-responsive healthcare. Cancer services contribute substantially to healthcare-related greenhouse gas emissions through energy consumption, transportation, pharmaceutical production, and medical waste generation. Strategies such as renewable energy utilization, energy-efficient hospital design, environmentally responsible procurement, sustainable pharmaceutical manufacturing, waste reduction, recycling programs, telemedicine, and optimized clinical pathways can reduce the environmental footprint of oncology without compromising patient outcomes. These initiatives align closely with SDG 13 while supporting broader Sustainable Development Goals related to health (SDG 3), sustainable cities (SDG 11), responsible consumption (SDG 12), and partnerships (SDG 17) (48). Although SDG 13 provides a valuable framework for addressing the environmental determinants of breast cancer, important evidence gaps remain. Few climate adaptation policies explicitly include cancer prevention indicators, environmental carcinogen surveillance, or resilience metrics specific to oncology services. Likewise, climate financing mechanisms have rarely prioritized research examining the relationship between environmental change and cancer. Future policy development should encourage interdisciplinary collaboration among climate scientists, oncologist s, environmental health researchers, epidemiologists, public health professionals, urban planners, economists, and policymakers to ensure that cancer prevention is incorporated into national climate action plans and health adaptation strategies (Figure 1) (49).

### Climate-related environmental exposures across breast cancer molecular subtypes

Breast cancer is a biologically heterogeneous disease comprising molecular subtypes with distinct genomic profiles, clinical behaviors, therapeutic responses, and prognoses. The major intrinsic subtypes include luminal A, luminal B, human epidermal growth factor receptor 2 (HER2)-enriched, and triple-negative breast cancer (TNBC), each characterized by unique patterns of hormone receptor expression and molecular signaling. Increasing evidence suggests that climate-related environmental exposures may not influence these subtypes uniformly because environmental toxicants interact with different biological pathways involved in tumor initiation and progression. Although definitive subtype-specific causal relationships remain incompletely established, recognizing this heterogeneity is essential for advancing precision environmental health and climate-responsive breast cancer prevention (48).

### Hormone receptor-positive breast cancer and endocrine-disrupting chemicals

Luminal breast cancers, particularly estrogen receptor-positive (ER-positive) tumors, account for approximately two-thirds of breast cancer cases worldwide. These tumors are highly dependent on estrogen signaling for cellular proliferation and survival. Climate change may increase human exposure to endocrine-disrupting chemicals (EDCs) through environmental contamination of air, water, soil, and food systems. Persistent pollutants such as bisphenol A (BPA), phthalates, polychlorinated biphenyls (PCBs), dioxins, organochlorine pesticides, and per- and polyfluoroalkyl substances (PFAS) possess estrogenic or anti-estrogenic properties capable of interfering with endocrine homeostasis (49). Experimental studies demonstrate that many EDCs activate estrogen receptor signaling, alter estrogen metabolism, promote mammary epithelial proliferation, induce epigenetic modifications, and disrupt normal mammary gland development. These mechanisms provide biological plausibility for an association between environmental exposures and hormone receptor-positive breast cancer. However, epidemiological findings remain inconsistent because exposure assessment, timing of exposure, genetic susceptibility, and cumulative lifetime exposures vary considerably across populations. Consequently, while endocrine disruption represents one of the strongest mechanistic links between climate-sensitive environmental exposures and ER-positive breast cancer, further prospective studies are required to establish causality (43, 50).

### HER2-positive breast cancer and environmental stressors

Compared with hormone receptor-positive disease, relatively few studies have specifically examined environmental determinants of HER2-positive breast cancer. HER2-enriched tumors are characterized by amplification of the ERBB2 gene and activation of proliferative signaling pathways, including PI3K/AKT and MAPK cascades. Environmental pollutants associated with oxidative stress and chronic inflammation may theoretically enhance these signaling pathways or promote genomic instability that contributes to HER2-driven carcinogenesis. Nevertheless, current evidence remains limited, and no consistent associations have been established between specific climate-related exposures and HER2-positive disease. This represents an important area for future molecular epidemiological investigation (51).

### Triple-negative breast cancer and inflammatory pathways

Triple-negative breast cancer (TNBC) lacks expression of estrogen receptor, progesterone receptor, and HER2, and is generally associated with aggressive clinical behavior, early metastasis, and fewer targeted therapeutic options. Unlike hormone receptor-positive tumors, TNBC is less likely to be influenced directly by endocrine signaling but may be more susceptible to environmental stressors that promote oxidative stress, chronic inflammation, immune dysregulation, and DNA damage (52). Climate-related air pollution, wildfire smoke, combustion-derived particulate matter, heavy metals, and persistent organic pollutants generate reactive oxygen species that induce genomic instability and inflammatory signaling within breast tissue. Activation of nuclear factor-kappa B (NF-κB), hypoxia-inducible factors, inflammasomes, and cytokine networks may create a pro-tumorigenic microenvironment capable of supporting aggressive tumor phenotypes. Although these mechanisms are biologically plausible, direct evidence linking climate-sensitive environmental exposures specifically to TNBC remains limited, and current conclusions should be interpreted cautiously (53).

### Epigenetic and immune mechanisms across molecular subtypes

Environmental exposures associated with climate change may influence breast carcinogenesis through epigenetic remodeling irrespective of molecular subtype. Air pollutants, endocrine disruptors, heavy metals, and persistent organic pollutants have been associated with altered DNA methylation, histone modifications, chromatin remodeling, and dysregulated microRNA expression. These epigenetic alterations may affect genes involved in cell-cycle regulation, DNA repair, apoptosis, immune surveillance, and hormone signaling, thereby contributing to tumor initiation and progression (54). Climate-related environmental stressors may also influence the tumor immune microenvironment. Chronic exposure to particulate matter and toxic chemicals has been associated with persistent low-grade inflammation, altered macrophage polarization, impaired natural killer cell function, T-cell dysfunction, and increased production of pro-inflammatory cytokines. Such immune alterations may contribute to tumor development across multiple breast cancer subtypes while potentially influencing therapeutic responses, particularly to immunotherapy. However, these relationships require further validation in clinical studies (55, 56).

### Life-course exposure and population susceptibility

The impact of climate-related environmental exposures is likely to vary according to the timing and duration of exposure. Critical windows of susceptibility—including prenatal development, puberty, pregnancy, lactation, and menopause—may represent periods during which environmental toxicants exert greater influence on mammary gland biology. Genetic predisposition, reproductive history, obesity, metabolic status, socioeconomic conditions, and coexisting environmental exposures further modify individual vulnerability. Consequently, breast cancer risk should be considered within a life-course exposome framework that integrates cumulative environmental, biological, behavioral, and social determinants rather than isolated risk factors (57).

### Climate change, social determinants of health, and breast cancer disparities

Breast cancer outcomes are shaped by a complex interplay of biological, environmental, behavioral, socioeconomic, and healthcare-related factors. While climate change has emerged as an important upstream determinant of health by modifying environmental exposures and disrupting healthcare systems, it should not be interpreted as the primary or sole driver of disparities in breast cancer incidence, stage at diagnosis, treatment outcomes, or survival. Rather, climate-related environmental exposures interact with well-established determinants of health, creating cumulative and often synergistic effects that disproportionately affect socially and economically disadvantaged populations (6). Marginalized communities—including individuals living in low-income settings, rural areas, informal settlements, Indigenous populations, and historically underserved racial and ethnic groups—often experience greater exposure to environmental hazards such as ambient air pollution, industrial emissions, contaminated water, wildfire smoke, and hazardous waste sites. Climate change may intensify these exposures through increasing temperatures, flooding, prolonged droughts, extreme weather events, and ecosystem degradation. However, environmental exposures represent only one component of a multifactorial framework influencing breast cancer outcomes (58).

Lifestyle-related risk factors remain major contributors to breast cancer burden and frequently coexist with environmental vulnerabilities. Obesity, physical inactivity, tobacco use, alcohol consumption, unhealthy dietary patterns, and metabolic disorders have well-established associations with breast cancer risk, particularly among postmenopausal women. Climate change may indirectly influence some of these factors by altering food systems, reducing opportunities for outdoor physical activity during extreme heat events, and increasing food insecurity, but these relationships are complex and are influenced by socioeconomic conditions, education, urban planning, and public health policies. Consequently, climate-related environmental changes should be considered as modifiers of established risk factors rather than independent explanations for observed disparities (59). Reproductive and hormonal characteristics also contribute substantially to breast cancer risk and vary across populations. Age at menarche, parity, breastfeeding practices, age at first childbirth, menopausal status, and use of hormonal therapies influence cumulative estrogen exposure and breast cancer susceptibility. These reproductive patterns differ according to cultural, demographic, and socioeconomic contexts and may partially explain variations in breast cancer incidence between populations. Current evidence does not support attributing these differences primarily to climate change, although environmental endocrine disruptors may interact with hormonal pathways in susceptible individuals (60).

Disparities in breast cancer outcomes are also strongly influenced by differences in access to preventive healthcare and timely diagnosis. Lower participation in mammography screening, delayed presentation, limited diagnostic capacity, inadequate pathology services, and restricted availability of specialized oncology care contribute significantly to more advanced disease at diagnosis in many underserved communities. Climate-related disasters—including floods, hurricanes, wildfires, and heatwaves—can further disrupt screening programs, damage healthcare infrastructure, interrupt transportation networks, and delay treatment initiation. Nevertheless, these disruptions often amplify pre-existing weaknesses within healthcare systems rather than acting as independent causes of healthcare inequity (61). Structural and systemic factors represent additional determinants of breast cancer disparities. Socioeconomic deprivation, limited health insurance coverage, educational inequalities, geographic barriers, workforce shortages, and racial or ethnic discrimination within healthcare systems have all been associated with delayed diagnosis, reduced treatment adherence, lower participation in clinical trials, and poorer survival outcomes. These inequities frequently coexist with greater environmental exposures, making it difficult to isolate the contribution of any single determinant. Therefore, an integrated framework that considers environmental, behavioral, biological, and structural determinants provides a more accurate understanding of breast cancer disparities than models focusing exclusively on climate-related exposures. The concept of the exposome offers a valuable framework for understanding these complex interactions by recognizing that breast cancer develops through the cumulative effects of environmental, behavioral, occupational, social, and biological exposures across the life course. Climate change influences this exposome by modifying environmental conditions, but its effects are mediated by individual susceptibility, genetic predisposition, reproductive history, lifestyle factors, socioeconomic position, and healthcare accessibility. Appreciating these interactions helps avoid oversimplified causal interpretations while supporting more comprehensive prevention strategies.

## Future research and translational opportunities

The relationship between climate change, environmental exposures, and breast cancer remains an emerging research field in which important biological and epidemiological questions remain unresolved. Future research should therefore move beyond demonstrating associations between individual environmental contaminants and breast cancer toward determining how climate-sensitive changes in exposure, biological susceptibility, social vulnerability, and healthcare disruption interact across the life course. Importantly, climate change should not be regarded as an established direct cause of breast cancer; rather, research should determine when and where climate change modifies environmental exposures or cancer-care pathways sufficiently to influence breast cancer risk and outcomes.

### Prospective climate–environment–breast cancer cohorts

Large prospective cohorts are needed to clarify temporal relationships between climate-sensitive exposures and breast cancer. Future studies should integrate longitudinal measurements of air pollution, wildfire smoke, temperature, pesticides, endocrine-disrupting chemicals, heavy metals, and other relevant exposures with detailed information on reproductive history, genetics, obesity, occupational exposures, socioeconomic status, and healthcare access. Repeated exposure measurements would be preferable to relying solely on residential location or historical averages. Long-term follow-up is particularly important because breast carcinogenesis may involve prolonged latency between exposure and clinical presentation. Prospective studies should therefore examine not only breast cancer incidence but also age at diagnosis, molecular subtype, stage, recurrence, treatment response, and survival.

### Life-course and windows-of-susceptibility research

Future investigations should examine environmental exposure across biologically sensitive periods, including prenatal development, childhood, puberty, pregnancy, lactation, and the menopausal transition. The mammary gland undergoes substantial structural and hormonal changes during these periods, potentially creating windows during which environmental exposures may have greater biological consequences. Such research should integrate environmental measurements with epigenetic, hormonal, metabolic, and immune biomarkers to determine whether early-life exposures produce persistent biological alterations that subsequently influence breast cancer susceptibility.

### Climate-sensitive exposure mixtures

Individuals are rarely exposed to a single pollutant. They experience complex mixtures of particulate matter, combustion products, pesticides, plastics-associated chemicals, metals, and other contaminants. Consequently, future studies should move from single-exposure models toward mixture-based exposure assessment. Advanced statistical approaches, including mixture modeling, Bayesian methods, exposome analysis, and causal-inference frameworks, could help identify combinations of exposures that are associated with breast cancer risk. Particular attention should be given to whether climate change alters the composition or intensity of these mixtures through wildfire activity, heat, flooding, drought, agricultural change, or chemical redistribution.

### Integration of environmental exposure with tumor biology

An important translational opportunity is to determine whether environmental exposures are associated with specific breast cancer phenotypes. Future research should investigate relationships between environmental exposure and:

- estrogen receptor status
- progesterone receptor status
- HER2 status
- triple-negative breast cancer
- tumor grade and stage
- genomic instability
- treatment resistance
- recurrence

This approach could determine whether environmental exposures influence particular biological subtypes rather than breast cancer uniformly.

### Multi-omics and exposomic research

Multi-omics represents a promising research strategy for investigating the biological consequences of environmental exposure. Integration of genomics, epigenomics, transcriptomics, proteomics, metabolomics, lipidomics, and microbiomics with detailed exposure data may reveal molecular pathways linking environmental conditions to breast carcinogenesis. However, these approaches should currently be regarded primarily as research tools rather than validated clinical tests for climate-associated breast cancer risk. Findings will require independent replication, biological validation, standardized exposure assessment, and prospective evaluation before translation into clinical practice.

### Environmental biomarkers and precision prevention

Future studies should evaluate biomarkers that can objectively characterize environmental exposure and biological response. Potential approaches include measurement of pollutant metabolites, persistent organic pollutants, metals, DNA adducts, oxidative-stress markers, hormone-disruption signatures, and epigenetic alterations. If validated, such biomarkers could complement established breast cancer risk factors and contribute to environmentally informed precision prevention. Nevertheless, clinical implementation should await evidence demonstrating that biomarker-guided interventions improve outcomes beyond established prevention and screening strategies.

### Artificial intelligence and environmental risk modeling

Artificial intelligence may provide an opportunity to integrate heterogeneous environmental and clinical datasets, including:

- air-quality measurements
- satellite-derived environmental information
- climate variables
- cancer registries
- electronic health records
- socioeconomic indicators
- occupational information
- molecular tumor characteristics

Machine-learning models could potentially identify populations experiencing unusually high environmental exposure or predict areas where climate-related disruption may threaten cancer services. At present, however, such applications should be considered investigational. Future studies should emphasize external validation, prospective evaluation, interpretability, calibration, data quality, and assessment of algorithmic bias. AI should complement rather than replace clinical judgment and established public-health surveillance.

### Remote sensing and geospatial exposure assessment

Satellite imagery, geographic information systems, and environmental monitoring networks may improve population-level assessment of wildfire smoke, air pollution, land-use change, temperature, industrial activity, and other environmental conditions. The translational value of these technologies will depend on their ability to generate reliable individual-level or community-level exposure estimates. Future studies should validate remotely sensed exposure measurements against ground-based monitoring and biological biomarkers before using them for clinical risk prediction.

### Environmental justice and vulnerable populations

Research should explicitly examine populations that experience disproportionate environmental and climate vulnerability. These include communities living near industrial facilities, mining areas, major transport corridors, agricultural zones, waste sites, or regions repeatedly affected by wildfire, flooding, drought, or extreme heat. Environmental exposure studies should incorporate socioeconomic conditions, healthcare accessibility, occupation, housing quality, energy sources, and geographic isolation. This will help determine whether climate-related environmental risks contribute to existing disparities in breast cancer incidence, stage at diagnosis, treatment completion, and survival.

### Research priorities in Africa and other climate-vulnerable regions

Evidence from Africa and other low- and middle-income regions remains insufficient relative to the magnitude of their climate vulnerability and cancer-control challenges. Future research should establish locally relevant exposure profiles encompassing biomass fuel use, ambient air pollution, mining, agricultural chemicals, industrial emissions, occupational exposures, extreme heat, and climate-related disasters. African breast cancer research should also integrate environmental exposure with the region’s distinctive demographic, reproductive, genetic, socioeconomic, and healthcare characteristics. Locally generated evidence is essential because exposure patterns and healthcare vulnerabilities cannot reliably be extrapolated from high-income countries.

## Conclusion

Climate change represents an emerging and multifaceted determinant of breast cancer risk, progression, and outcomes. By altering environmental exposures, exacerbating pollutant burdens, disrupting healthcare access, and amplifying social inequities, climate-driven stressors influence both the biological and systemic pathways that shape breast cancer trajectories. Vulnerable populations—particularly women in low-income, rural, or marginalized communities—bear a disproportionate burden, highlighting the intersection of environmental injustice and health disparities.

Integrating climate considerations into breast cancer prevention, care, and policy aligns closely with the objectives of SDG 13—Climate Action. Measures that reduce emissions, regulate endocrine-disrupting chemicals, and strengthen climate-resilient health systems offer co-benefits for both environmental sustainability and cancer control. Cross-sectoral collaboration, adaptive infrastructure, and climate-informed surveillance are essential to ensure equitable access to screening, timely diagnosis, and uninterrupted treatment. As climate change accelerates, its influence on breast cancer will likely intensify, necessitating proactive and integrated strategies. A climate-aware oncology framework that incorporates environmental stewardship, health-system resilience, and population equity is essential to mitigate risk, improve outcomes, and ensure that progress in breast cancer control is sustainable in a changing global landscape.
